# Randomised controlled effectiveness study (RCT) of isometric exercise (IE) in adults with stage 1 and 2 hypertension – ISOFITTER study

**DOI:** 10.3310/nihropenres.14059.3

**Published:** 2026-04-14

**Authors:** Melanie Rees-Roberts, Ellie Santer, Rachel Borthwick, Timothy Doulton, Pauline A Swift, Tracy Pellatt-Higgins, Katerina Gousia, Douglas MacInnes, Alan West, John Darby, Anusree Biswas, Caroline Cowley, Christoher K Farmer, Jonathan Wiles

**Affiliations:** 1 Centre for Health Services Studies, University of Kent, Canterbury, England, CT2 7NZ, UK; 2 East Kent Hospitals University NHS Foundation Trust, Canterbury, England, CT1 3NG, UK; 3 Epsom and St Helier University Hospitals NHS Trust, Carshalton, Surrey, SM5 1AA, UK; 4 Personal Social Services Research Unit, University of Kent School of Social Policy Sociology and Social Research, Canterbury, England, UK; 5 Canterbury Christ Church University, Canterbury, England, CT1 1QU, UK; 6 Public co-applicants, Canterbuy, UK

**Keywords:** Hypertension, blood pressure, isometric exercise, lifestyle intervention, wall squat, prevention

## Abstract

Background High blood pressure (BP) affects more than one in four adults in England and only one in three patients are being treated effectively. Treatment of high BP includes changes to lifestyle such as more physical activity and/or taking medication. However, low adoption and high attrition are common with the large amounts of exercise currently recommended (>150 minutes moderate exercise per week plus 2 strength sessions). Evidence suggests that isometric exercise (IE), holding a fixed body position for a period of time, for example a wall squat, results in greater long term BP reductions, with less time and effort, than other recommended exercise. This ISOFITTER study aims to evaluate the effectiveness of IE to help reduce BP in hypertension in a real world setting. Methods A multi-centre, randomised, controlled trial of an isometric exercise wall squat intervention for hypertension employing an effectiveness-implementation hybrid type-1 design. Adults (n = 542) with Stage 1 or Stage 2 hypertension, on no more than one antihypertensive, and no other medical contra-indications will be randomised to either a standard care plus IE intervention group or standard care control group. Blood pressure readings, fidelity measurements, medications, adverse events, quality of life, participant satisfaction and health service use will be collected at baseline, week 4, month 3 and month 6 with a subgroup (n = 50) of the IE arm invited to continue for 12 months. Qualitative participant focus groups and interviews with wider stakeholders will collect implementation data. Results The ISOFITTER study will establish effectiveness of a self-administered, home-based IE intervention in lowering blood pressure in people with uncomplicated stage 1 and 2 hypertension. Implementation evidence will support patient delivery, context for scaling up of the intervention and intervention cost. Conclusion Lifestyle changes for the treatment of hypertension in the absence of other risk factors should not be overlooked. For long term hypertension management, easily adopted, evidenced exercise interventions are needed. This study will help to address this evidence gap.

## Plain Language Summary

This research aims to see if a wall-squat isometric exercise training programme (leaning with your back against the wall in a sitting position) will reduce peoples blood pressure for those diagnosed with high blood pressure.

**Background to the research:**

High blood pressure affects more than one in four adults in England and only one in three of these successfully reduce their blood pressure through treatment. The cost of this to the NHS is high (approximately £2.1 billion per year), mainly due to care related to strokes and heart attacks that can be caused by high blood pressure. Treatment of high blood pressure includes changes to lifestyle such as healthy eating and exercise and/or taking medication. A type of exercise, called isometric exercise, involves holding a fixed body position for a set amount of time. This type of exercise includes things like leaning with your back against the wall in a seated position and has been shown to be better at lowering blood pressure than other types of exercise like running. This study follows on from smaller research projects to see if a wall-squat isometric exercise is good for lowering blood pressure as it was found to be easily done at home to help lower blood pressure. Healthcare professionals also thought it was a good exercise option for those with high blood pressure. This larger study is now needed to be confident that this isometric exercise can reduce people's blood pressure reliably, at home.

**Study design:**

This study has been designed with public advisors and will involve 542 adults with a high blood pressure diagnosis, without any other significant health conditions. We will use a wide approach to asking people to take part, including those from less represented backgrounds. The study will be advertised through social media, community groups, health services and GP surgeries. All 542 people that take part will receive healthy lifestyle advice. Half of those taking part will then be randomly chosen to do the isometric exercise and the other half with be a control group and asked not to change anything. This control group will be offered the exercise after 6 months. The isometric exercise plan involves completing three wall-squat sessions per week for six months. All those taking part will record their blood pressure and other lifestyle information at four-weeks, three-months and six-months so we can see whether the exercise has made a difference.

## Introduction

High blood pressure (BP) or hypertension in the UK is defined as a clinic BP of ≥140/90 mmHg or a home BP measurement ≥135/85 mmHg and it is a leading modifiable risk factor for morbidity and mortality. ^1^ ^,^ ^2^ Hypertension may be diagnosed based on a clinic BP >140/90 confirmed by out-of-office >135/85 using either averaged home readings or ambulatory blood pressure monitoring (ABPM). BP above optimal levels (>120/80 mmHg) are linearly associated with an increased risk of cardiovascular disease (CVD) and is responsible for approximately 75,000 deaths in the UK. ^3^ ^,^ ^4^ It has been estimated that over ten years, 45,000 quality adjusted life years and £850 m could be saved if England and Wales achieved a 5 mmHg reduction in population systolic BP. ^5^

An estimated 32% of adults in England have high BP with three in ten of those (4.2 million) undiagnosed. ^6^ Alarmingly, data indicate that a much higher proportion of younger adults (16 to 24) with the condition remain undiagnosed compared to those aged 75+. ^7^ Adults from black minority ethnic groups are more likely to be diagnosed with hypertension. ^8^ Hypertension prevalence varies with deprivation, from 23% to 40% in the most deprived. ^9^ The United Kingdom’s (UK) Chief Medical Officer’s report (2021) emphasised increased prevalence of hypertension and cardiovascular mortality in coastal and deprived regions. ^10^ It is a top priority of the Core20PLUS5 National Health Service (NHS) strategy for reducing health inequalities across England. ^11^

National UK guidance for the treatment of hypertension is two-tiered: initial lifestyle advice (diet, weight management, exercise, reduction in alcohol and salt intake) and then anti-hypertensive medication if BP remains elevated (if clinic BP is >180/120 mmHg at first encounter or is subsequently found to be ≥150/95 on home or ambulatory BP monitoring antihypertensive medication is recommended alongside lifestyle change). ^12^ Only 40–50% of treated hypertensives achieve target BP mainly due to poor adherence, lack of efficacy of anti-hypertensives or lack of tolerability. Therefore, there is urgent need for treatments to replace or complement anti-hypertensives without compounding undesirable side effects of pharmacotherapy, often deemed unacceptable, especially to younger age groups. ^13^

**Figure 1.**
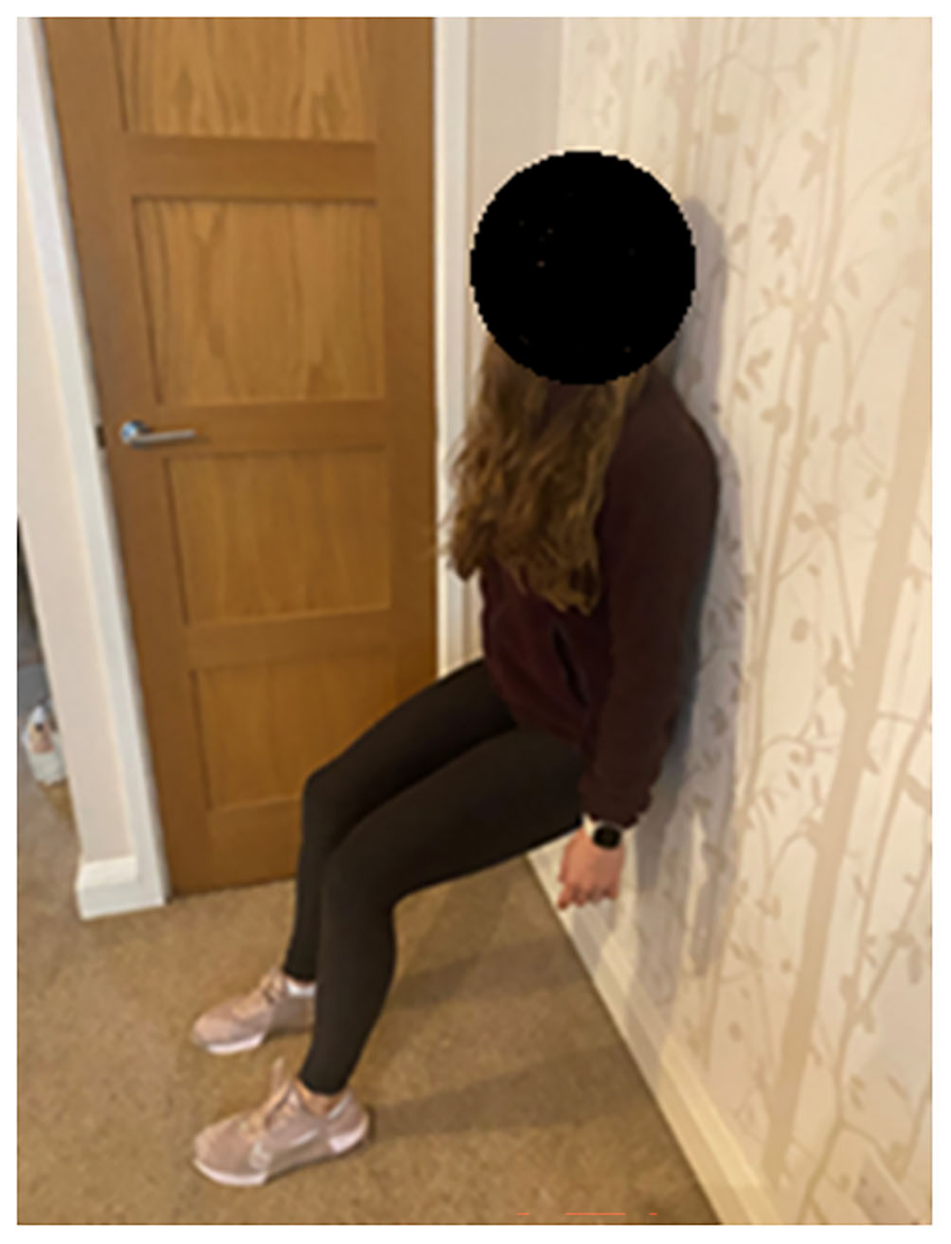
Person performing the wall squat exercise.

The importance of lifestyle changes in hypertension treatment in the absence of other risk factors should not be overlooked. ^1^ Exercise is associated with anti-hypertensive benefits and can be as effective as medication in controlling BP. ^14^ ^–^ ^17^ However, low adoption and high attrition rates are common. ^14–18^ To promote lifestyle exercise changes, those with hypertension need easily adopted, effective and manageable exercise interventions as a first line option. Meta-analyses indicate that isometric exercise (IE) results in larger reductions in BP compared to more traditional forms of exercise ^15^ and has great potential to treat hypertension, ^19^ improve health and reduce mortality. Isometric exercise can lower BP in people with both normal ^20^ and high-normal BP (pre-hypertensive) ^21^ and is a potentially effective lifestyle intervention for hypertension. IE involves holding a fixed position for a period of time: muscles are used but there is no movement, e.g. leaning against a wall in a seated position ( Figure 1). Only 24 minutes of IE a week are required to achieve reductions in BP of 12/6 mmHg in pre-hypertensives, which can be easily carried out at home without costly equipment. ^21^

Our feasibility study demonstrated that an IE wall-squat is deliverable, acceptable to patients and professionals, has low dropout and no adverse effects. ^22^ This article describes the protocol for the ISOFITTER study, a follow on randomised controlled clinical trial to determine the effectiveness and implementation potential of individually tailored IE training, at home for people with Stage 1 and 2 hypertension.

## Trial design and setting

**Figure 2.**
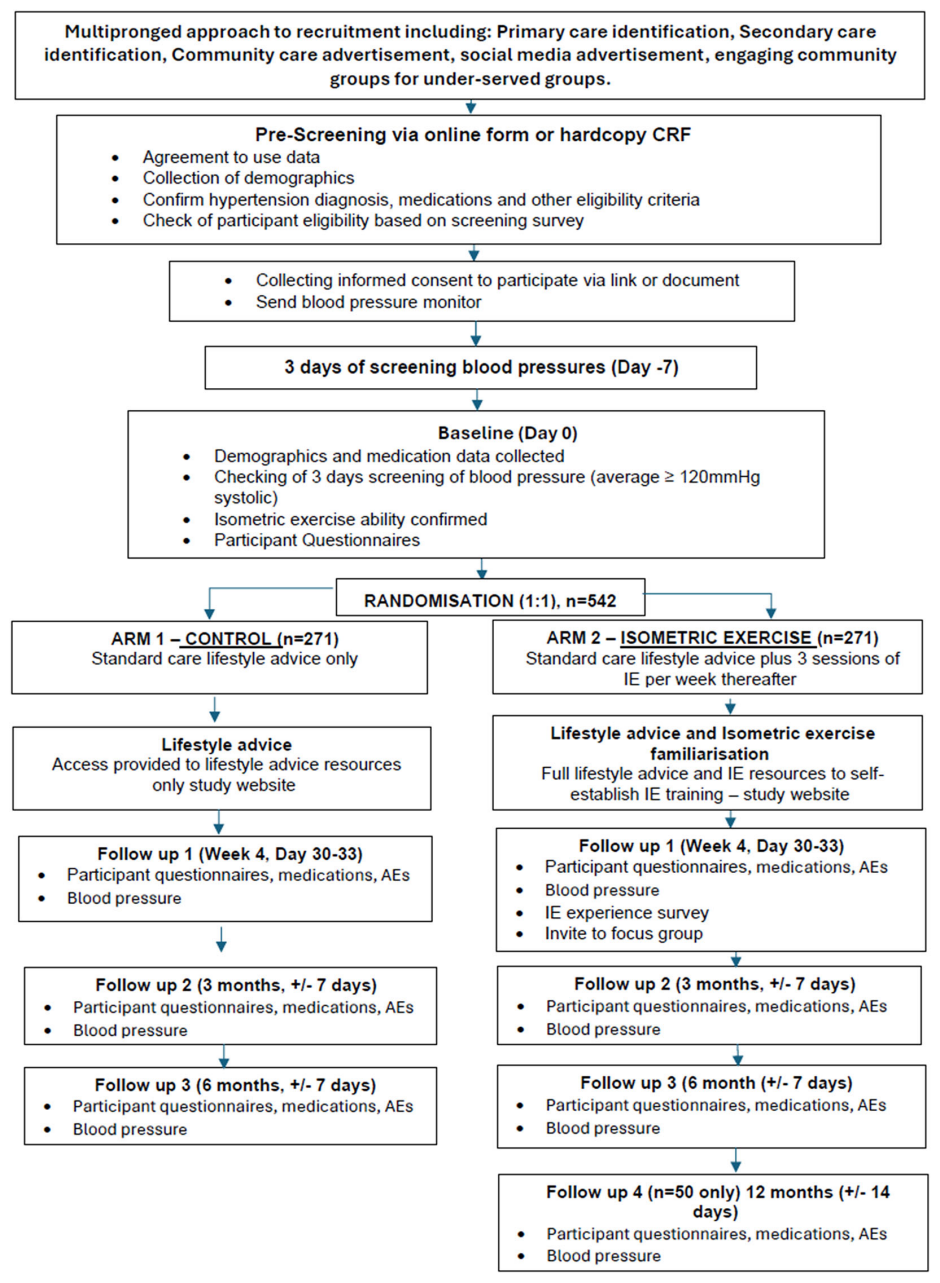
ISOFITTER Study Flowchart.

This study is a multi-centre randomised, controlled trial (RCT) of an isometric exercise intervention for participants with Stage 1 and Stage 2 hypertension following an effectiveness-implementation hybrid type-1 design. ^23^ The RCT will involve parallel study groups of standard care plus IE (intervention group) versus standard care alone (control group). Figure 2 presents a flowchart of the study design. The study will be delivered direct to public through advertising and primary care participant identification centres across England and Wales. Our geographical areas of focus are East Midlands, Yorkshire and Humber, South London, South-East England and Wales.

## Study objectives

The aim of the ISOFITTER study is to determine the effectiveness of self-prescribed IE at home in addition to standard care for reducing systolic BP in people with Stage 1 and 2 hypertension and understand contexts for implementation.

Primary objective: to determine the effectiveness of IE in reducing systolic BP in people with stage 1 and 2 hypertension compared to standard care.

Secondary objective: to understand relevant implementation contexts that support the ability to take up the intervention from a participant, provider/commissioner and cost perspective to explore:

• participant acceptability, adoption and feasibility, relevant to experiences in the ability to take up, self-administer/conduct the IE programme, and barriers and facilitators for long-term adherence;
• healthcare provider and stakeholder perspectives (including commissioners) of sustainability and implementation into standard care pathways;
• cost consequence analysis to provide a range of cost information to support commissioning decisions from outcomes.

## Protocol

The current version of the ISOFITTER protocol at the time of publication was Version 2, 15 ^th^ January 2025.

### Participant eligibility criteria

Participants included in the study must be aged 18+ with stage 1 or stage 2 hypertension (defined as a clinic BP of ≥140/90–179/119 mmHg diagnosed by a health care professional and confirmed by a GP). Participants should be prescribed no more than 1 antihypertensive agent and have no significant medical condition that would contraindicate their participation. They must be able to undertake an IE intervention and able to self-monitor BP.

A significant medical condition that would contraindicate participation outside of the exclusion criteria below is any medical condition declared by the participant or identified by the recommending physician which may affect a participant’s safety during the study (e.g. significant knee pain or weakness, epilepsy, narcolepsy etc).

Exclusion criteria include: alteration to antihypertensive medications within 6 weeks prior to screening (either dose or number of medications); receiving a β-blocker; averaged home systolic BP <120 mmHg following 3 days screening, ischaemic heart disease, stroke or transient ischaemic attack in the past 3-months; moderate or severe valvular heart disease, atrial or ventricular arrhythmia, congenital or inherited heart condition; pregnancy or actively trying to conceive; enrolled in Clinical Trial of Investigational Medicinal Products (CTIMP)/device/other interventional study of BP; locomotor abnormality resulting in inability to do wall squat or any condition that would be made worse by doing the wall squat exercise.

Exclusion of participants who were taking beta adrenergic blocking agents (beta-blockers) is due to attenuated heart rate, blood pressure and haemodynamic responses which would potentially undermine efficacy when prescribing this as well as other forms of exercise to lower resting blood pressure. Whilst this approach may exclude a proportion of people with hypertension treated, we think this would be relatively few. This is because our study only includes people on a single antihypertensive agent and beta-blockers are fourth-line agents in the treatment of uncomplicated hypertension according to NICE guidelines. ^1^

### Sample size

Variability was estimated from the feasibility study. ^24^ Estimates of the standard deviation (SD) for systolic BP change from baseline at 4-weeks, 3 and 6-months were 12.5, 10.8 and 14.4 mmHg respectively. With a minimal clinical important difference 5 mmHg and SD of 14.4, we require 352 participants to complete this study to attain 90% power with a 5% significance level. Assuming 35% attrition we aim to randomise 542 participants, 271 in each group. The sample size confidence interval has been calculated using Pass11 software (PASS 11. NCSS, LLC. Kaysville, Utah, USA. www.ncss.com).

For qualitative data samples, we will conduct up to four online focus groups of 6–8 participants each from the intervention group plus up to 15 interviews with stakeholders – it is expected that this sample will achieve data saturation whereby no new qualitative data codes/elements are identified when approaching the full sample. ^25^

### Participant recruitment

This study will utilise recruitment through up to 20 General Practice participant identification centres (PICs), using direct to public advertising through posters, public flyers, media including social media, associated partners and charities (e.g. Healthcare providers, Blood Pressure UK, British and Irish Hypertension society) and through outreach activities for under-represented groups. Demographics of participants recruited will be closely monitored across age, sex, disability, ethnicity and socioeconomic status to identify any under-represented groups against an expected representative sample calculated from UK hypertension prevalence data and UK population data. This will help guide targeting of under-represented groups and achieving a representative sample as discussed below. All participants will be directed to a pre-screening online form in the form of an advertisement or flyer with QR code or direct link. In GP recruitment PICs, a standardised search will identify potentially eligible participants from their medical records who will receive a text message with a link to the pre-screening form online. These searches and eligibility screening tasks will be done by the direct care team, or where appropriate by the National Institute for Health and Care Research (NIHR) regional RDN agile team. For outreach recruitment, participants may complete the pre-screening form in person with a member of the research team or wider partner organisations and events.

To achieve a representative sample of the UK population, we will enhance recruitment strategies and adverts to facilitate a balanced recruitment to include ethnicity, gender, age and level of deprivation. Recruitment through GP PICs with diverse ethnic groups and from a range of indices of deprivation will be recruited. We will include voluntary questions during screening including ethnicity, sexual orientation, gender, socioeconomic status and disability to monitor the recruitment strategy. A Research Inclusion Committee (see oversight groups) will review the profile of the study population during the recruitment period and advise on missing groups and best methods for addressing imbalances in the study sample. The group will advise on outreach recruitment and public engagement activities in underserved and under-represented populations such as working with smaller or umbrella charities/organisations representing particular populations (e.g., barber shops, hairdressers, opticians, religious organisations/leaders, supermarkets, post-natal groups and large employment organisations).

### Participant consent

All participants in this study are required to provide informed consent prior to any interventions or assessments. Pre-screening eligibility form data is collected anonymously and permission to use unidentified data entered in the online form is confirmed in the pre-screening form prior to full informed consent. This allows important screening demographic information to be collected anonymously, including date of birth, gender, ethnicity, socioeconomic status, level of education and sexual orientation. These data will help to understand and monitor the diversity of participants interested in taking part in the study. Data for all those who complete the pre-screening form, regardless of whether they are eligible or not, will be retained to inform purposeful recruitment strategies to reach underserved groups, unless the participant does not agree to their data being used in this way. Where the participant is eligible at pre-screening and enters their contact details, their demographic information will be linked to their study ID number and future data automatically upon full study consent to avoid duplication of data collection at baseline.

Only participants with capacity will be included in the study. The study will be delivered remotely, and informed consent is completed by the participant online after receiving the online full information sheet and after online pre-screening to determine eligibility. Where needed the informed consent form can be emailed/posted as well as the option to go through consent with a member of the study team if they wish for accessibility. Support is available if participants require assistance to consent online or via paper consent. Informed consent is collected in the secure online research electronic data capture (see data management section). Where interested participants are identified in person or at outreach events, consent can be taken at these in person events with appropriate countersignature. Alternatively, they can complete informed consent at a later date either with the study team or recruitment site teams to consent using our alternative options (as above). Once a participant has consented to the study, they will be allocated a unique Participant Identification Number, a copy of the electronic consent form or the paper form (where used) will be provided to the participant (by email or post) and a copy held centrally in the Trial Master File.

For qualitative data collection, study participants are invited to take part in a focus group at Week 4 (see qualitative data collection section below) and written informed consent is collected online through the same data capture software. A copy of the informed consent is emailed to the participant ahead of the focus group if selected. For stakeholder interviews, verbal informed consent is sought and recorded prior to starting the interview. Verbal informed consent has been chosen to minimise the administrative burden on time constrained health and care professionals and maximise chances of recruitment.

### Participant enrolment

***Pre-Screening and Participant Information***

An online pre-screening eligibility form with questions based on the study inclusion and exclusion criteria including: age, their blood pressure (confirmed by their GP), all medications, antihypertensive medication, medication changes within the last 6 weeks, if they experienced any of the excluding conditions in the past 3-months, pregnant and whether they are enrolled in another CTIMP/device/other interventional study of BP. If pre-screening eligibility is confirmed, participants are notified that they appear to be eligible for the study and are presented with a short summary of the study. They then have the option to enter their email address to receive the full study information. If identified as eligible, participants will be sent a maximum of two email reminders if they do not sign up directly after pre-screening.

The participant is then sent via email, text or post, a Participant Information Sheet (PIS) and link to the online consent form. The study website displays a downloadable PIS for participants and they will also be able to request the PIS by post or email if they prefer. For other recruitment routes where potentially eligible participants are recruited in person (e.g. outreach events), copies of the PIS will be available. The study website will also have details of a dedicated telephone, text and email address of the delivery team where interested participants can ask further questions or for the information to be read to them. At GP recruitment PICs, in-person support may also be available to discuss the study. Where translation into other languages may be needed, participants will be signposted to translate the information documents online (via Google). Those interested will be prompted to take sufficient time (ideally at least 24 hours) to consider the information prior to consenting to take part. Participants will also be encouraged to discuss taking part in the study with their GP if needed.

Further contact details (name, postal address, email confirmation, mobile number) as well as GP name and address are collected at the end of the consent process in order facilitate:

• sending out automated blood pressure devices for participant screening and beyond;
• sending out of study documents if requested or required for accessibility reasons;
• sending of a GP letter to participant’s GP practice to inform them of study participation and what this involves;
• setting up access to participant portal online with digital resources including instructional videos;
• mobile and email reminders of study assessments and training if assigned to IE group.

***Screening***

After successfully completing pre-screening and upon receipt of their home blood pressure monitor, they will be instructed via email to measure their blood pressure for 3 days consecutively at home, taking 3 measurements in the morning and evening according to European Hypertension Society and NICE guidelines. ^1^ ^,^ ^26^ Measurements will be conducted using the home BP device (Kinetik Wellbeing WBP1 model number TMB-1775-A and model TMB-2287-K for those requiring an extra-large cuff ) provided by the study which will be mailed to the participant along with an instruction sheet and participant diary. Participants are instructed to continue normal activities during the screening period. Participants are made aware in the PIS and again prior to entering their screening data that a final decision about their eligibility to participate in the study after collection of:

• past medical history (in relation to eligibility criteria)
• current medication
• 3 days of home blood pressure measurements
• confirmation of ability to do IE

The calculation of blood pressure for entry into the study is conducted using the 2nd and 3rd reading on the 3 consecutive days morning and evening (total of 12 readings used to calculate the average home blood pressure). The average of all readings for home blood pressure is conducted to ensure we aren’t including people with either overtreated hypertension, white coat hypertension or people mis-diagnosed as hypertensive. Eligibility is then assessed against the full criteria. Those who screen fail will be sent an email thanking them for their contribution to the study and provided with website links for maintaining a healthy blood pressure and lifestyle (the same standard care advice provided to all participants in the study). Those who pass screening then proceed to randomisation and enrolment in the study as below.

***Randomisation***

Where participants screening data is assessed as making them eligible, participants will be randomised to either a standard care control group, or standard care plus 3 sessions of IE per week intervention group. Participants will be randomised in a 1:1 ratio using pseudo-random numbers. Random permuted blocks will be used within stratification to conceal the sequence of treatments and ensure that treatments are balanced at the end of every strata block. Stratified randomisation will ensure balanced groups based on age (≤50, >50 years), biological sex at birth (male, female), and treatment status (hypertension medication, no hypertension medication), giving 8 strata in total. Participants will be allocated using an online data capture software (see data management section).

As an exercise intervention study, participants will not be blinded to their group allocation. An independent statistician was employed to ensure that the study statistician will be blinded to randomisation allocations and will only analyse unidentifiable data. Data will only be unblinded in the case of serious adverse events (see page 13–14) and for the Chief Investigators only. The study statistician will not receive the 12-month data until the main study data has been analysed, and treatment allocation revealed. At the end of the study, all participants will have access to the IE exercise programme content, including control participants should they wish to try the IE programme and as an incentive to continue as a control in the study until then.

***Study Intervention***

The participant’s normal care provider (GP or hospital where appropriate) will retain responsibility for the participant’s healthcare outside the research study. On completion of the baseline data collection (see Participant timeline and assessments below), participants will be provided with access to relevant intervention and/or standard care resources within the study website portal and via email. The resources will also include an overview of the timepoints, data collection and training for specific measurements (such as blood pressure monitoring). There will be a dedicated telephone support line during office hours. Participants will be encouraged to continue their normal activities during the study.

***Standard care advice – healthy lifestyle – all participants***

Standard care lifestyle advice consists of National Institute for Health and Care Excellence (NICE) guidance with or without a single antihypertensive agent (excluding beta-blockers due to impact on heart rate and possible attenuation of the benefit of IE). ^1^ Standard care advice will be provided in the online study portal and by email to all participants as a video summary and signposting to the BPUK “How to Lower Your Blood Pressure” webpage. ^27^

***Isometric Exercise Training – intervention arm only***

The intervention will be self-delivered by participants using the instruction materials provided (videos) within the website portal and written instructions (downloadable or by email/post if requested). Participants will be taught how to perform an isometric exercise wall squat ( Figure 1) ^28^ that is tailored to their own optimum intensity that should be conducted for four bouts of two minutes each in the appropriate squat position. Two minutes rest will be completed in between each bout. ^21–30^ This will be performed three times a week for the duration of the study a total of 6-months (or 12-months for a sub-group of n = 50).

**Figure 3.**
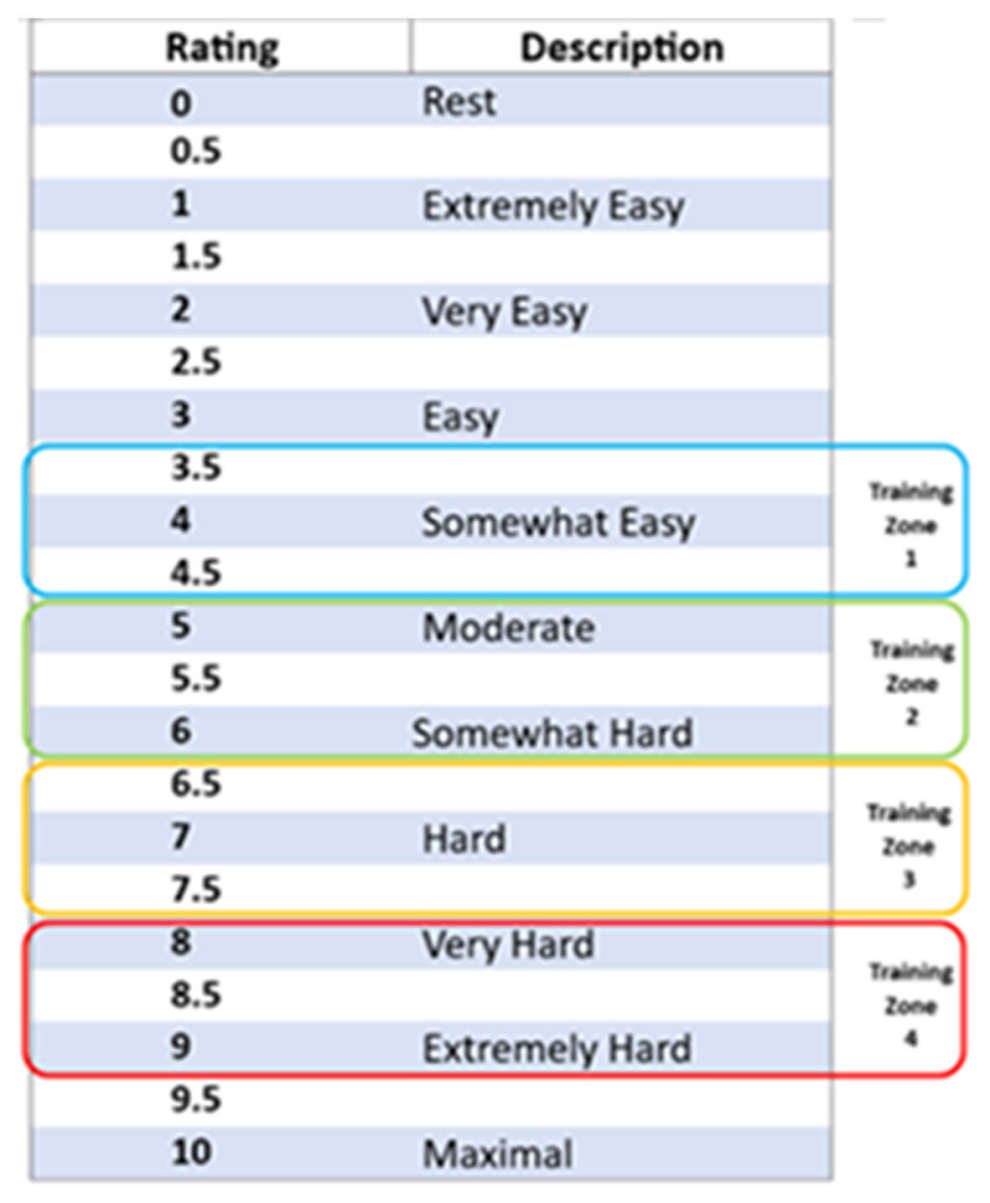
Rating of Perceived Exertion (RPE) scale for optimising training intensity.

Personalisation of the exercise plan is achieved in the first session by establishing an individualised optimum intensity ^31^ based on a rating of perceived exertion (RPE) for isometric exercise scale ( Figure 3). ^32^ ^,^ ^33^ The isometric wall squat protocol employed uses RPE to determine the correct individualised wall squat height/knee joint angle to elicit the necessary level of cardiovascular stress proven to result in longer term resting blood pressure reduction/adaptation. ^31^ ^,^ ^32^ The progressive RPE training zones used to inform the four bouts that make up each isometric exercise session have been determined to replicate a warm up period and achieve a target heart rate and blood pressure previously documented when using the originally validated wall squat protocol based upon 95% HRpeak as measured during an incremental isometric exercise test. ^20^ ^,^ ^21^

How high the participant’s back is positioned against the wall affects the perception of exertion when performing an isometric wall squat and this can be used to personalise their back position/squat height for optimum training sessions. Initially, participants perform a 30-second wall squat to locate an approximate height that they estimate would result in a score of 4 on the RPE scale when held for 2-minutes ( Figure 2 – training zone 1) – this will then be marked on the wall using white tack or a marker. Following 2-minutes of seated rest, this is repeated for four bouts of 2-minutes IE with 2-minutes rest in between, feeling harder each time. Each time they will rate their perceived exertion and whether this falls within the correct training zones ( Figure 2 training zones 1–4). If outside the target range, they will be instructed to alter their squat position up or down by 1 cm to achieve target range. At the end of this set of wall squat positions participants will have established and marked the optimum depth of their individual wall squat and will be achieving the appropriate physiological response for clinical benefit. ^31^ ^–^ ^33^

Intensity is maintained and monitored by RPE ratings during subsequent training sessions. If a participants RPE in the final bout of any session is not in target zone 4, they are advised to adjust their wall-squat position up or down by 1 cm to maintain optimum training intensity going forward. To check fidelity of the intervention early on in the study an interim analysis of RPE recorded at the last bout of the first 3 exercise sessions for the first 50 participants in the intervention arm will be conducted.

### Participant on study timeline and assessments

This section describes the assessments and visits conducted with participants once they are randomised. Table 1 overleaf summarises the schedule of assessments at each time point and Figure 2 provides a flow chart of participant flow through the study. There are no prohibited interventions during the trial. Participants’ (control and intervention) GP’s or other relevant healthcare organisation (e.g. hospital) will continue to be responsible for normal care provision with respect to hypertension and other medical conditions through and post study participation. A letter will be sent to the participant’s GP to inform them of participation in the study and what that involves. Participants will be able to request a copy of this. Participants will be advised to seek advice and care from their GP throughout the study should their condition change. Participants will be asked not to alter medication unless absolutely necessary and after consultation with their GP. Participants with BP > 180/120 will be advised to seek urgent medical review, GPs will be advised to follow NICE guidelines in relation to urgent specialist referral.

**Table 1.** Participant schedule of assessments.

Assessment	Pre- screening	Screening	Baseline Day 1 (start date of IE training)	Follow up 1 Day 30 (+/− 3 days)	Follow up 2 Month 3 (+/− 7 days)	Follow up 3 Month 6 (+/− 7 days)	Follow up 4 ^1^ Month 12 (+/− 7 days)
Pre-screening form	X						
Collection of unidentified demographic data	X						
Consent		X					
Home BP readings		X ^2^	X ^3^	X	X	X	X
Confirmed IE ability		X					
Randomisation			X				
Standard Care lifestyle advice			X				
Self-prescribing of IE ^4^			X				
Outside range RPE data ^5^				X			
Diet questionnaire			X		X	X	X
Concomitant medication			X	X	X	X	X
Exercise questionnaire			X		X	X	X
Quality of life questionnaire			X		X	X	
IE experience questionnaire				X			
Health resource use questionnaire					X	X	
Invitation to take part in study focus group				X			

1 – first n=50 invited and accepting to continue follow up until Month 12 2 - recorded between Day -7 and Day 0 3 - if Screening BP measures ≥28 days prior to baseline 4 - IE group only 5 – collected from the first n=50 participants only for fidelity assessment

[i] 1 – first n = 50 invited and accepting to continue follow up until Month 12 2 - recorded between Day −7 and Day 0 3 - if Screening BP measures ≥28 days prior to baseline 4 - IE group only 5 – collected from the first n = 50 participants only for fidelity assessment.

***Baseline assessment***

Once the participant has been randomised, they will receive an email providing details of how to set up their log in details and enter the study website portal where they will have access to standard care lifestyle advice resources and IE exercise training videos for those in the intervention group. Participants will be instructed to start their standard care advice and IE training if assigned, at a time when they have at least 60 minutes to run through the information and are requested to complete the initial standard care advice and IE training induction within 14 days of completing the baseline questionnaire and/or no more than 28 days after they finished their 3 days of home BP readings. If not completed within this time, one final reminder will be sent before they are withdrawn from the study. If a randomised participant does not complete their standard care advice and IE training within 14 days, they will be able to start this for up to 3 months after consenting to take part, however after 14 days they will be asked to confirm that their eligibility has not changed.

***Follow up assessments – Week 4, Month 3, Month 6 and Month 12***

All participants will be followed up at week 4 (Day 30 +/−3), at months 3 and 6 (+/− 7 days). Follow up at month 12 will be conducted for a sub-group of the first n = 50 intervention group participants to evaluate longer term BP changes. This longer term, 12-month follow up is restricted to 50 participants due to the funding envelope and duration of 30 months which allows the research to explore longer term outcomes within limited time and funding whilst maintaining value for money. An invitation will be sent to the participants up to 2 weeks before finishing their 6 months of IE, to invite them to continue to 12 months and the reasons why. Invitations will be sent until there is an uptake of 50 for the subgroup.

Participants will receive electronic reminders (email, text or WhatsApp message depending on participant preference) ahead of each follow up time point to prompt data collection. They will receive further reminders on the day of assessment and then daily during the submission window (refer to above) to submit their information through the online questionnaire. Intervention arm participants will receive reminders of exercise sessions as per their preferences set during baseline data collection. Weekly motivational messages will also be sent to control participants for the first 6 weeks of the study. This will aid adherence to the study and IE training and reminders will include a link to input their data each time or at regular weekly intervals. At each follow up, participants will record their home blood pressure, heart rate readings and in their participant diary and submit these via an online questionnaire using a link emailed or texted to participants. Alternative methods for data recording will be made available to participants where requested, for accessibility (telephone call, on paper etc.) and supported by local teams or the study team centrally.

The protocol requires three consecutive measurements (separated by two minutes) always using the same arm (use arm with high reading if >15 mmHg difference when first checked), to be taken in the morning and afternoon for three days at each timepoint. Home blood pressure monitoring will follow NICE and ESH/ESC guidelines ^1^ ^,^ ^26^ using a British and Irish Hypertension Society approved blood pressure monitor. Participants will be asked to keep their chosen times of blood pressure measurements and ingestion of medication consistent throughout the study. Adverse events, checking of medication, adapted version of UK EPIC NORFOLK Food Frequency Questionnaire, ^34^ General Practice Physical Activity Questionnaire, ^35^ EuroQOL quality of life 5D-5L questionnaire, ^36^ study-specific IE intervention experience questionnaire (intervention group only at week 4) and health economics data (questionnaire adapted for the study from ^37^) will also be collected according to the schedule of assessments ( Table 1). To confirm fidelity of the intervention, data about whether RPE scores were outside of range and alteration to their wall squat was required from the first n = 50 IE participants will assess if training is being conducted as intended. At the end of all follow ups, participants will be asked to return any paper records to the study team in a stamped, addressed envelope provided in their study pack.

***Qualitative data collection***

Qualitative data will be collected from participants and stakeholders interested in sharing their experiences of taking up, self-administering and conducting the intervention as well as understanding barriers and enablers for long-term adherence. This work will provide information on data on intervention feasibility, acceptability, adherence and implementation. These data will inform and refine the intervention approach of direct to patient delivery, e.g. referral pathways, clarity of instructions and self-administration, experience of ongoing adherence. The information will also inform the implementation of the intervention on a wider scale if effective and the relevant context considerations, mechanisms for access, rationale for future commissioning and conditions in which this is best achieved.

***Participant focus groups***

We will conduct up to four online focus groups of 6–8 participants each from the intervention group, the structure and conduct of which will be guided by Krueger and Casey’s work. ^38^ An invitation to take part in a participant focus group will be sent to intervention group participants after the week 4 assessment completion. Participants will then be able to register interest via an online link and expressions of interest pooled until sufficient numbers to start the focus groups. If over-subscribed, we will select enough to participate from this pool, ensuring participants are as representative as possible of the population of England and Wales. Participants will be purposively selected to ensure diversity including geographical location, socio-economic status and relevant protected characteristics. Those who are selected to participate will be contacted to confirm their place, those who are not selected will be informed that they will not need to attend a focus group and thanked for volunteering.

Focus group participants will be asked for permission to digitally record the conversations, after full information including the fact that any comments made will not be directly attributable. The focus groups are proposed to last for 60–90 minutes and will be undertaken remotely via video call (MS Teams). Those who require support in accessing the online call will be provided with bespoke support to take part. The topic guide used in the feasibility study will be adapted to the objectives of this follow on qualitative sub-study, working with our lay co-applicants who supported development of the initial guides. Lay co-applicants will be invited to help co-facilitate focus groups for which they will receive appropriate training.

***Stakeholder interviews***

Healthcare professionals and stakeholders will be invited to take part in individual interviews (n = 15) by email advertising through research team contacts, NIHR Applied Research Collaborations and Health Determinant Research Centres, the Health Innovation network, Research Delivery Network, professional bodies, collaborating charities and other networks. Where any healthcare professional group is under-represented, further email invitations sent out will be targeted accordingly to obtain the views of those key stakeholders. Individual interviews are the preferred method for this group as interviews can be set up at a convenient time. Interviews will explore perspectives of implementation using an interview guide informed by relevant Consolidated Framework for Implementation Research ^39^ constructs exploring contexts for successful implementation of the IE intervention. Interviews are expected to take between 45 and 90 minutes and will be conducted on the telephone or video call (MS teams). Participants will receive a short summary of the intervention, with study preliminary findings ahead of interview to inform discussion. Therefore, interviews will be scheduled to take place towards the end of the project. Participants will be asked to verbally consent to the interview and it’s recording thereafter.

***Ancillary and post-trial care***

The study will be completed when the last participant has completed their last visit, either Month 6 (for most participants) or Month 12 (long term follow up sub-group). After the last participant, last assessment, study data will be cleaned before the database is locked for analysis. At the point of each participant completing the study, they will receive an email thanking them for their participation. Participants in the control arm will be offered access to the IE training programme content after their last visit. All participants will also be allowed to retain the BP monitor equipment provided to facilitate ongoing health monitoring and as an incentive for participation. A lay summary of the results will be shared with study participants as soon as possible via the study website or hardcopy via local delivery teams.

At the end of their participation, normal standard care through health services will continue as it has during the study.

### Statistical methods

The primary outcome will be to assess a change from baseline home average systolic BP (mmHg) in the IE intervention arm compared to control arm at 3-months. The secondary outcomes to be evaluated at 4-weeks, 3-months, 6-months include:

• proportion of participants with home average BP of ≤135/85 mmHg compared to baseline;
• prescription changes from baseline to timepoints using anti-hypertensive treatment intensity score ^40^;
• home average Systolic BP at 4-weeks, 6-months in IE vs Control and 12-months in IE;
• home average Diastolic BP at 4-weeks, 3-months, 6-months in IE vs. control and 12-months in IE;
• evidence the fidelity of the study intervention when it is self-prescribed (RPE recorded in range at the last bout for the first week of training) from a subset of the first 50 participants in intervention arm;
• understand participant experiences of ability to take up, self-administer and conduct the IE programme, including barriers and facilitators for long term adherence;
• changes in the use and costs from a UK NHS and personal social services perspective in both groups at 3-months and 6-months; changes in the QOL in both groups at 3-months and 6-months;
• total cost of the intervention and cost per participant; cost consequence analysis of the intervention including QALYs in both groups at 3-months and 6-months;
• to evaluate other changes in lifestyle because of uptake of IE as a lifestyle measure at 3 months, 6 months and 12 months in IE.

The study was powered to detect a 5 mmHg difference which is deemed to be representative of the minimal clinical important difference. A subset (n = 50) of participants will be followed to 12-months where consent is obtained.

Quantitative data analysis.

The primary outcome, change from baseline in systolic BP at 3-months, will be analysed using analysis of covariance, with a fixed treatment effect allowing adjustments for baseline values, sex, age, and hypertension medication status. The primary analysis will be conducted using an intention to treat approach with a two-sided 5% significance level. Secondary analysis will examine treatment effects under different scenarios for compliance with allocation/treatment, using per protocol approaches. Prior to analysis distributional assumptions will be assessed, and the data may be transformed or alternative nonparametric approaches used where there is evidence of non-normality. Data from the IE experience questionnaires will be analysed using descriptive statistics, as will secondary process outcomes such as recruitment and adherence rates, attrition and completeness of the data.

We will undertake an interim analysis of the first 50 participants’ fourth bout RPE scores and the extent to which these are in range of the target zone to check intervention fidelity to determine the extent to which the intervention is being delivered as intended. Data collected for this purpose involves confirmation from participants on whether they altered their wall squat height and whether their RPE was outside of range.

To avoid loss of efficiency, missing outcome values (both primary systolic blood pressure and secondary diastolic blood pressure) will be imputed using multiple imputation, if the proportion of missing data is greater than 5% and less than 40%. ^41^ Where there are less than 5% missing data, the proportion of missing data is considered negligible and missing observations will be excluded. Multiple imputation methods perform less well when the amount of missing data is substantial, if more than 40% of the primary outcome data are missing in the primary analysis the assumptions are less plausible. The interpretative limitations of the trial data will be discussed in the results section, where this is the case.

An imputation model containing all potential prognostic baseline covariates, the primary outcome variable at 4 weeks follow-up, and the secondary outcome variable diastolic blood pressure at 4 weeks follow-up will be used. ^42^ The number of imputations will be dependent on the amount of missing data, as a minimum the number of imputations will be derived to ensure at least 96% statistical efficiency (RE) according to the formula below, where *λ* is the fraction of missing values and M is the number of repetitions. ^42^

The statistical model and assumptions made in the analysis of the primary outcome will also be implemented in the multiple imputation procedures. If it is suspected data is missing not at random or the pattern of missing data is associated with trial allocation, sensitivity analysis will be performed using a pattern mixture approach with mixed modelling and multiple imputation ^5^ to compare the sensitivity of conclusions to varying assumptions about the missing value mechanism. All available data from baseline to the time of dropout will be included in the sensitivity analysis using a repeated measures mixed effects model.

A full Data Analysis Plan can be found in our study Data Repository referenced at the end of this manuscript in the Data Availability section.

***Qualitative data analysis***

Inductive thematic analysis of focus group/interview transcripts will be carried out using NVivo 11. Thematic analysis will be carried out using Braun and Clarke’s (2013) six stage model. ^43^ Drawing on Sweeney *et al*.’s ^44^ notion that the service user researcher unique perspective should be preserved rather than subsumed, the process will involve multiple members (including public co-applicants) independently (stages 1–3, data familiarisation, initial coding and themes) before joining together to generate a consensus view (stages 4–6, reviewing, defining, naming themes and report production). To guide the thematic analysis of the participant, focus groups, a behaviour change framework (COM-B) has been used to refine the interview questions and will be used to guide analysis. ^45^ For stakeholder interviews, the RE-AIM framework will be used to understand full implementation contexts. ^46^ The findings will be fed back to respondents for validation or revision of the interpretations. Deviant case analysis will be used to ensure that perspectives that diverged from dominant trends are not overlooked.

***Health economic analyses***

We will conduct a cost consequence analysis and present evidence on a range of costs and outcomes associated with the intervention. We will present the number and percentage of participants with missing values for each cost element and each outcome measure. Data from the QOL and economic questionnaires will be transferred to STATA 18 and analysed using descriptive statistics. We will estimate the mean and 95% confidence intervals for each type of cost (intervention, NHS primary care, NHS secondary care), and the difference between intervention and control group. We will calculate the mean QOL and 95% confidence intervals for both groups both unadjusted and after adjusting for baseline utilities and we will estimate the difference between the two groups. We will also estimate the QALYs for each group at 3-months and 6-months.

### Study governance

***Participant withdrawal***

Participants are free to withdraw at any time from the study without giving reasons and without prejudicing their further treatment. They will be made aware of this during consent and at each study timepoint. They will also be informed that data collected up to the point of withdrawal will be used unless they specifically state that they do not wish for this. The right of a participant to refuse participation without giving reasons will be respected.

***Trial registration***

The study is registered with the UK’s clinical study registry, the ISRCTN under reference number 28188474.

***Study sponsorship***

East Kent Hospitals University NHS Foundation Trust are the sponsor and are responsible for ensuring regulatory compliance, trial monitoring, safety, proper data management, and that the trial is adequately funded and concluded. Name and contact information for the trial sponsor:

East Kent Hospitals University NHS Foundation Trust.

Caroline Cowley.

Research & Innovation/Clinical Trials Unit Manager caroline.cowley@nhs.net

***Ethical approvals***

Ethical approval has been granted by the National Health Service Health Research Authority London - Bromley Research Ethics Committee (Reference number 24/LO/0911, IRAS number 331559). The study will be conducted in accordance with the UK Policy Framework for Health and Social Care Research March 2018 ^47^ and recommendations for physicians involved in research on human subjects adopted by the 18th World Medical Assembly, Helsinki 1964 and later revisions. ^48^

***Protocol amendments***

Protocol amendments will be decided on by the project team and Chief Investigators. Full version control procedures will be adopted. Protocol amendments will be submitted and approved by the ethics committee where appropriate before being put in place.

***Study oversight groups***

The ISOFITTER study steering committee is chaired by Professor Indranil Dasgupta (Chair of the British and Irish Hypertension Society Collaborative Research Standing Committee). The steering committee is composed of at least 75% members who are independent to the study team. The committee includes the Chief Investigators, an independent statistician and health economist, clinicians with expertise in hypertension/clinical trials, an exercise specialist, an expert in qualitative health services research, an implementation specialist and two independent lay members. The committee critically oversees progress, outputs, deliverables and governance of the project. The delivery of the study is managed by the East Kent Clinical Trials Unit (CTU). A project management group brings together the CTU and the academic delivery team from the University of Kent and Canterbury Christ Church University. A research inclusion subcommittee, chaired by Dr Anusree Biswas Sasidharan as a public co-applicant on the study, oversees recruitment of a representative study sample, ensuring inclusion of participant groups commonly under-represented in research such as those on low income and those from minority ethnic groups.

***Adverse event monitoring***

For the purpose of this study an adverse event (AE) is defined as any untoward medical occurrence in a study participant, whereas a serious adverse event (SAE) is any untoward medical occurrence that results in death, is life-threatening at the time of the event, requires participant hospitalisation or prolongation of existing hospitalisation, results in persistent or significant disability/incapacity, or consists of a congenital anomaly or birth defect. Other ‘important medical events’ may also be considered serious if they jeopardise the participant or require an intervention to prevent one of the above consequences. Only AEs or SAEs which might have resulted (definitely or probably) from the study intervention, e.g. muscular injury in the leg as a result of IE, will be recorded. These will be submitted by the participant in their assessment questionnaires. The CTU will monitor reported AEs from participants and report any AEs or SAEs as following standard governance processes and requirements.

Common wall squat related adverse events would be similar in nature to other forms of recommended exercise (e.g. aerobic exercise or strength training) and may include muscle soreness after exercise, stress on joints (particularly the knees and ankles), dizziness during or after exercise, blood pressure spikes if holding of breath occurs during exercise (instructions to breath normally is including in the instructions to participants) and potential post-exercise hypotension. These will be recorded throughout the study.

In the event of an Adverse Event (AE) or Serious Adverse Event (SAE) the clinical lead applicant would be unblinded to the participant’s randomisation group to allow them to assess the relatedness of the event to the intervention. This information would be available on the data collection questionnaire/forms stored centrally in the trial master file. This information would be shared by the delivery team only when necessary.

Although IE can be undertaken in pregnancy, we have decided to take a conservative approach and exclude pregnant participants. Any women of childbearing potential should agree to use a medically accepted method of contraception while they are participating in the study. Should a participant get pregnant during the study, they will be asked to notify the study team within 7 days of becoming aware of the pregnancy. In case of a pregnancy while receiving study treatment, a participant should be withdrawn from the study.

***Data monitoring and audit***

Only the CTU delivery team will have access to the full dataset throughout the study. The statistician and research team will have access to an anonymised data set for analysis. Upon completion of analysis, the anonymised data will be available to other researchers upon reasonable written request with clearly defined purpose and its use will be to benefit of the wider society (see section 7).

Study data will be collected and managed using REDCap electronic data capture tools ^49^ ^,^ ^50^ hosted at East Kent Clinical Trials Unit. All data will be entered on to a secure server using REDCap software. Data quality will be enhanced using range checks and prompts for missing fields. The decision not to establish a Data Monitoring Committee (DMC) for this project is because the project has been evaluated as low risk by the Sponsor’s Risk Assessment. This included an evaluation of participant safety with the interventions/procedures involved being considered to pose minimal risk to participant health and well-being, the inclusion of relatively healthy participants who are treated or untreated for hypertension, and finally the disease under study does not involve high-risk or complex conditions that would require intensive monitoring to ensure safety or efficacy. Ongoing data for this project are reported monthly to the Project Management Group to review data to support the progress of the study and to address any emerging issues or concerns. Given this structured and regular oversight, the need for a separate, external DMC is not deemed necessary.

Data will be reported on a quarterly basis to the Trial Steering Committee for review. Central/remote monitoring activities will take place monthly from first recruit onwards. Remote monitoring activities will be as follows, independent of the investigators and carried out by CTU Governance and/or Facilitator:

• informed consent completed on REDCap reviewed – first participant and 10% monthly;
• investigator site file review;
• training & delegation review;
• informed consent process documentation for consent received over the telephone.

To protect the participants, minimal personally identifiable information will be collected. The participant will input their data directly onto the data management system REDCap. Only those members of the study team with delegated duties will have access to these data. Participants agree to share contact details with the BP machine provider and the courier who will deliver the BP monitor. Contact details held by the delivery team will be destroyed at the end of the study, retaining only participant study codes. Only non-identifiable information can be shared with the wider study team for the purpose of the project. Any personal data will be stored within REDCap software or password-protected computers within a limited access folder on the Sponsor network with unique codes allocated to each service user.

Personal data will only be shared with the university partners for the purpose of contacting participants directly about their exercise intervention or for participation in participant focus groups. Electronic files with personal information will be password protected and stored on the university partner networks in folders that can only be accessed by the research team. These folders will not be transferred from the network onto personal computers. Data in paper form will be transferred into electronic copy and destroyed as early as possible, but where it needs to be stored this will be in locked filing cabinets in offices that are locked when unoccupied. Access to the data collected during the project (including any participant personal data) will be restricted to the research team, and data will not be shared with anyone else. At the end of the study the data will be electronically archived by the sponsor and destroyed after five years.

***Dissemination policy***

Participants and stakeholders will receive an easy read summary of the results which will also be shared widely on mainstream and social media. It will also be available for use by our collaborating and other national charities and professional bodies to disseminate findings. Reporting to the funder will be 6-monthly and a final report at study completion. Publication will be in peer-reviewed journals, including an effectiveness data paper, a separate implementation contexts paper and a commentary on the approaches to representative recruitment. There will be an executive summary of study findings for providers and commissioners and healthcare local organisations. Findings will be presented at national and international conferences, e.g. European Hypertension Society and British and Irish Hypertension Society. Standard International Committee of Medical Journal Editors (ICMJE) authorship guidelines will be adhered to and there will be no use of professional writers in any publications. The IE training protocol will be made publicly available online at the end of the study.

## Public and patient involvement

The study is being delivered using co-production principles with members of the public with or without lived experience of hypertension. Three public co-applicants have been involved with the study from early design stages, including methodology and objectives, through to set up and delivery. Contributions included co-designing the participant intervention resources, co-design of the study website, decisions on recruitment strategies for inclusivity and reviewing of this protocol and other study related documentation. An additional two public members sit on the Trial Steering Committee. A further group of public members assist with ad hoc requests and document reviews. The public advisory team continue to support with reviewing study processes, documentation, recruitment and ongoing management including monitoring risks. Furthermore, they will support with the participant focus groups, qualitative analysis and review of lay summaries. Our public co-applicants also sit on our Research Inclusion Committee, chaired by one of the public co-applicants chairs this 6-weekly panel.

## Conclusion

The ISOFITTER study will establish whether self-administered, home-based wall squat isometric exercise in addition to standard lifestyle advice lowers blood pressure by 5 mmHg in people with uncomplicated stage 1 and 2 hypertension with or without a single blood pressure agent. It will provide implementation evidence to support patients to adhere over the long term, context for scaling up of the intervention and its cost.

## Consent to publish

Consent to publish a persons image in Figure 1 has been obtained for the purposes of publishing this article. No other identifiable information is included in this article.

## Data Availability

On study completion and publication, access to an anonymised data set will be available where the sharing of data has a clearly defined purpose and its use will be of benefit to wider society in line with UK policies. ^51^ When available, data can be found in the study external repository which is in the public domain ^52^. Data that will be made available includes:
o Anonymised quantitative study data where participants have consented to use for future research o Qualitative study data where participants have opted in to the use of their data for future research. Open Science Framework. Randomised controlled effectiveness study of isometric exercise (IE) in adults with stage 1 and 2 hypertension (ISOFITTER study). Project identifier: DOI http://dx.doi.org/10.17605/OSF.IO/QHFBW Dataset weblink: OSF | Randomised controlled effectiveness study of isometric exercise (IE) in adults with stage 1 and 2 hypertension (ISOFITTER study) ^48^ This project contains the following underlying data:
• Protocol paper supplementary documents – contains the SPIRIT 2 checklist for protocol paper publication. • Study documentation: Contains all study documentation including protocol, participant facing documents and data collection tools • Study Data Analysis Plan • On study completion, this repository will contain a folder with fully anonymised study data once analysis is completed. Data is available under the terms of the CC-BY 4 International license.

## Reporting guidelines

This protocol complies with the Standard Protocol Items: Recommendations for Interventional Trials checklist (SPIRIT checklist). A copy of the checklist with reference to sections of this protocol paper is included in supplementary documents.
