# Supplementary material for: Randomised controlled effectiveness study (RCT) of isometric exercise (IE) in adults with stage 1 and 2 hypertension – ISOFITTER study
Source: NIHR Open Res. 2026 Apr 14;5:78. Originally published 2025 Sep 11. [Version 3] doi: 10.3310/nihropenres.14059.3 (PMC12869024; doi:10.3310/nihropenres.14059.3)
Supplement: Peer review history

# Reviewer report: Reviewer response for version 2

**DOI:** 10.3310/nihropenres.15456.r38463

**Published:** 2026-02-03

**Authors:** Gavin Devereux

**Affiliations:** 1 University of Suffolk, Ipswich, UK

The ISOFITTER study is a multi-centre randomised controlled trial evaluating the effectiveness of a self-administered, home-based isometric wall squat exercise programme for reducing blood pressure in adults with stage 1 or 2 hypertension. A total of 542 participants receiving standard care, and taking no more than one antihypertensive medication, will be allocated to either standard care alone or standard care plus the isometric exercise intervention. Outcomes including blood pressure, quality of life, medication use, adverse events and healthcare utilisation will be measured over six months, with a subgroup followed for up to 12 months, alongside qualitative assessments to explore implementation. The study aims to provide robust evidence that time-efficient isometric exercise can offer a practical, scalable and effective lifestyle-based approach to improving long-term hypertension management.

 The background and rationale are clear in terms of the main sections of the submission, and I believe the design of the study is appropriate. The methods detail is also clear.

 While there are instances where I would question the syntax used, my overall assessment is largely subjective and open to debate and may reflect personal preference. However, I consider the Abstract to be an exception, given its prominence and importance to readers. For example, on Line 3 of the Background, "large target" does not read very well when associated with a prescription of duration of time. And, at the end of the Background section, the final sentence does not read very well. In fact, it read as though IE will create hypertension.

Is the study design appropriate for the research question?

Yes

Is the rationale for, and objectives of, the study clearly described?

Yes

Are sufficient details of the methods provided to allow replication by others?

Yes

Are the datasets clearly presented in a useable and accessible format?

Not applicable

Reviewer Expertise:

I previously researched the effects of isometric exercise on resting blood pressure, and the potential mechanisms that may explain reductions in resting blood pressure after a period of isometric exercise training.

I confirm that I have read this submission and believe that I have an appropriate level of expertise to confirm that it is of an acceptable scientific standard.

# Response

**Published:** 2026-03-23

**Authors:** Melanie Rees-Roberts

**Affiliations:** CHSS, University of Kent, Canterbury, England, UK

Dear reviewer, thank you for your comments and taking the time to peer review this article. We have updated the abstract to reflect your important points and to make it clearer to the read in relation to describing the study accurately.

# Reviewer report: Reviewer response for version 1

**DOI:** 10.3310/nihropenres.15289.r38469

**Published:** 2025-11-25

**Authors:** Heriviyatno J Siagian

**Affiliations:** 1 University of Sembilanbelas November Kolaka, Kolaka, Indonesia

**Overall Assessment**

 The protocol is well written, comprehensive, and based on robust preliminary work. The rationale is clinically relevant given the widespread burden of hypertension and challenges in exercise adherence among adults. The design is appropriate, the intervention is clearly defined, and ethical procedures are adequately addressed. However, several methodological clarifications would improve transparency and reproducibility.

**Major Points**

 1. Clarification of Handling of Missing BP Data

 The protocol relies heavily on participant-reported home BP readings. However, the procedures for handling missing or incomplete BP readings, particularly at multiple time points, are not fully described.

*Recommendation:*

 Include a clear plan for missing data management (e.g., multiple imputation, last observation carried forward, complete case analysis, sensitivity analyses).

 2. More Detail Needed on Adherence Monitoring

 Although reminders and participant diaries are described, objective adherence monitoring for the intervention group is limited.

*Recommendation:*

 Specify whether:

- digital logs from the website will track video access or completion of training sessions,
- self-reported adherence will be validated by any secondary method,
- adherence thresholds will be defined for per-protocol analysis.

*3. Screening Process Needs Clearer Description*

 Participants perform 3-day home BP monitoring to determine eligibility, but the protocol does not specify:

- what happens when morning and evening readings differ significantly,
- whether an average of all readings or a subset is used for eligibility confirmation,
- criteria for excluding participants with high variability.
  Recommendation:
  Clarify the exact algorithm used to derive final eligibility BP and criteria for exclusion based on variability or measurement error.

4. Implementation Outcomes Require Defined Framework

 Implementation elements are mentioned (acceptability, adoption, feasibility), but the protocol could benefit from explicitly mapping these to established frameworks (e.g., CFIR, RE-AIM).

 Recommendation:

 Define which constructs will guide qualitative data analysis and how these map onto secondary objectives.

**Minor Points**

1. Flowchart (Figure 2)
  - Consider simplifying the visual flow to improve readability for readers unfamiliar with hybrid designs.
2. Training Personalization Procedure
  - The description of wall-squat height adjustment is clear but could benefit from a brief rationale explaining how RPE correlates with physiological response.
3. Diversity and Inclusion Plan
  - The recruitment strategy is commendably comprehensive; however, operational definitions of "under-represented groups" could be more explicit.
4. Safety Considerations
  - The protocol rightly excludes individuals with contraindications. Including a brief summary of potential wall-squat-related risks (e.g., muscle strain, dizziness) would strengthen the safety section.
5. Data Sharing Section
  - It may be helpful to specify whether anonymised qualitative data will also be available upon request.

Is the study design appropriate for the research question?

Yes

Is the rationale for, and objectives of, the study clearly described?

Yes

Are sufficient details of the methods provided to allow replication by others?

Partly

Are the datasets clearly presented in a useable and accessible format?

Not applicable

Reviewer Expertise:

Hypertension; Kidney Diseases

I confirm that I have read this submission and believe that I have an appropriate level of expertise to confirm that it is of an acceptable scientific standard, however I have significant reservations, as outlined above.

# Response

**Published:** 2025-12-08

**Authors:** Melanie Rees-Roberts

**Affiliations:** CHSS, University of Kent, Canterbury, England, UK

Dear reviewer,

 Many thanks for taking the time to read and comment on this paper. The authors would like to thank the peer reviewers for their helpful and constructive comments in reviewing this protocol paper. The following changes have been made in response to these comments:

Reviewer 2 (25 Nov 2025)

1. *Clarification of Handling of Missing BP Data: The protocol relies heavily on participant-reported home BP readings. However, the procedures for handling missing or incomplete BP readings, particularly at multiple time points, are not fully described. Recommendation: Include a clear plan for missing data management (e.g., multiple imputation, last observation carried forward, complete case analysis, sensitivity analyses).*

**Response: **Thank you for this request, we have included the below short description in the Quantitative analysis section to clarify how we will handle missing data.

 ‘To avoid loss of efficiency, missing outcome values (both primary systolic blood pressure and secondary diastolic blood pressure) will be imputed using multiple imputation, if the proportion of missing data is greater than 5% and less than 40% (41). Where there are less than 5% missing data, the proportion of missing data is considered negligible and missing observations will be excluded. Multiple imputation methods perform less well when the amount of missing data is substantial, if more than 40% of the primary outcome data are missing in the primary analysis the assumptions are less plausible. The interpretative limitations of the trial data will be discussed in the results section, where this is the case.

 An imputation model containing all potential prognostic baseline covariates, the primary outcome variable at 4 weeks follow-up, and the secondary outcome variable diastolic blood pressure at 4 weeks follow-up will be used (42). The number of imputations will be dependent on the amount of missing data, as a minimum the number of imputations will be derived to ensure at least 96% statistical efficiency (RE) according to the formula below, where l is the fraction of missing values and M is the number of repetitions (42).

 The statistical model and assumptions made in the analysis of the primary outcome will also be implemented in the multiple imputation procedures. If it is suspected data is missing not at random or the pattern of missing data is associated with trial allocation, sensitivity analysis will be performed using a pattern mixture approach with mixed modelling and multiple imputation [5] to compare the sensitivity of conclusions to varying assumptions about the missing value mechanism. All available data from baseline to the time of dropout will be included in the sensitivity analysis using a repeated measures mixed effects model.  

 A full Data Analysis Plan can be found in our study Data Repository referenced at the end of this manuscript in the Data Availability section.’

 A full project Data Analysis Plan has also now been finalised and can be found open access in our data repository for the study for transparency.

1. *More Detail Needed on Adherence Monitoring. Although reminders and participant diaries are described, objective adherence monitoring for the intervention group is limited. Recommendation: Specify whether:*

- *digital logs from the website will track video access or completion of training sessions,*
- *self-reported adherence will be validated by any secondary method,*
- *adherence thresholds will be defined for per-protocol analysis.*

**Response: **As this is a pragmatic study and due to funding constraints, digital logs of the website to track video access are not being collected. Qualitative focus groups with participants in the intervention arm will allow us to explore the usability and barriers/facilitators to the study resources that enable isometric exercise programmes to be established by participants and how much they used them.

 To confirm fidelity of the intervention, the study has a planned early interim analysis consisting of RPE recorded at the last bout of the first 3 exercise sessions for the first 50 participants in the intervention arm to check whether participants are reporting within the target training zone. A sentence has been added to the Isometric exercise intervention section to clarify this as follows:

 ‘To check fidelity of the intervention early on in the study an interim analysis of RPE recorded at the last bout of the first 3 exercise sessions for the first 50 participants in the intervention arm will be conducted.’

 At each follow up timepoint, participants are asked to confirm the number of sessions completed in the month prior to the timepoint to check completion of training sessions. This will be used to determine our per-protocol analysis. The per protocol data will contain all data for participants who complete the trial as planned – in intervention and standard care groups - without any major protocol violations or exclusions. Per protocol analyses essentially drop those individuals who have not complied with their allocated treatment – both those who only partially complied with their allocated treatment (less than 2/3 sessions in the past month prior to the timepoint) and those who did not receive their allocated treatment. This means that per protocol analysis will represent a likely ‘best case scenario’ for treatment effect estimation.

 The study will also complete an intention to treat analysis containing all available data for participants who were randomised, regardless of whether or not they complied with allocation. This dataset will include participants who were withdrawn/withdrew from the trial post-randomisation.  These analyses are a lower bound estimate of treatment effects as they represent the effect of offering a programme, rather than the effect of receiving that programme.

 We also plan an exploratory analysis to see if our data may elucidate the minimum number of training sessions that elicit clinical benefit on blood pressure. We have detailed both these analysis approaches in the Quantitative data analysis section and we have now added reference to the study Data Analysis Plan which will be made available in the study data repository for transparency.

  

1. *Screening Process Needs Clearer Description. Participants perform 3-day home BP monitoring to determine eligibility, but the protocol does not specify:*

- *what happens when morning and evening readings differ significantly,*
- *whether an average of all readings or a subset is used for eligibility confirmation,*
- *criteria for excluding participants with high variability.*

*Recommendation: Clarify the exact algorithm used to derive final eligibility BP and criteria for exclusion based on variability or measurement error.*

**Response: **To determine eligibility, UK and European guidelines are used to determine the blood pressure at screening which acts as one key criteria for proceeding to randomisation and enrolment in the study. This is conducted for two reasons, firstly to exclude white coat hypertension and secondly to confirm blood pressure is within the range stated for inclusion criteria and classifies as Stage 1 or Stage 2 hypertension. At screening three sets of systolic and diastolic blood pressure measurements are requested morning and evening for 3 consecutive days (total of 18 measurements). To calculate eligibility, the first of each set is discarded, the remaining two from each day, morning and evening, are combined and averaged to provide the blood pressure used to assess against study eligibility criteria. As a stringent criterion are set at screening with multiple measurements to account for day to day and within day variability, all subsequent analyses will be conducted on the average of all blood pressure readings recorded at baseline and follow up timepoints. This is detailed in the Data Analysis Plan which is now available on our study data repository. We have added the following to the screening section to give more detail on this:

 ‘The calculation of blood pressure for entry into the study is conducted using the 2 ^nd^ and 3 ^rd^ reading on the 3 consecutive days morning and evening (total of 12 readings used to calculate the average home blood pressure). The average of all readings for home blood pressure is conducted to ensure we aren't including people with either overtreated hypertension, white coat hypertension or people mis-diagnosed as hypertensive. Eligibility is then assessed against the full criteria.’

1. *Implementation Outcomes Require Defined Framework. Implementation elements are mentioned (acceptability, adoption, feasibility), but the protocol could benefit from explicitly mapping these to established frameworks (e.g., CFIR, RE-AIM). Recommendation: Define which constructs will guide qualitative data analysis and how these map onto secondary objectives.*

**Response: **To refine our qualitative data collection instruments from our feasibility study, we have used the following frameworks:

- Participant feasibility and acceptability – the COM-B behaviour change framework
- Stakeholder acceptability, adoption and feasibility – the Re-AIM framework.

These frameworks will be used to guide inductive thematic analysis of the qualitative data. To clarify this, we have added the following including new references for the frameworks into the qualitative data analysis section:

 ‘To guide the thematic analysis of the participant, focus groups, a behaviour change framework (COM-B) has been used to refine the interview questions and will be used to guide analysis (45). For stakeholder interviews, the RE-AIM framework will be used to understand full implementation contexts (46).’

1. *Flowchart (Figure 2). Consider simplifying the visual flow to improve readability for readers unfamiliar with hybrid designs.*

**Response: **We have used a standard flow chart template for the visual flow of the hybrid study design. We feel this is sufficient and has been reviewed during development of the protocol by the full research team including public members who did not raise any issues with readability of the visual flow. No changes made to this.

1. *Training Personalization Procedure. The description of wall-squat height adjustment is clear but could benefit from a brief rationale explaining how RPE correlates with physiological response.*

**Response: **To provide more detail on how the RPE correlates with physiological response, we have included the below paragraph in the Intervention section:

 ‘The isometric wall squat prescription protocol employed on the ISOFITTER study uses rating of perceived exertion (RPE) to determine the correct individualised wall squat height / knee joint angle to elicit the necessary level of cardiovascular stress proven to result in longer term resting blood pressure reduction / adaptation (Lea et al. 2023). The progressive RPE training zones used to inform the four bouts that make up each isometric exercise session have been determined to replicate a warm up period and achieve a target heart rate and blood pressure previously documented when using the originally validated wall squat protocol based upon 95% HRpeak as measured during an incremental isometric exercise test (Wiles et al 2017, Taylor et al 2019).’

1. *Diversity and Inclusion Plan. The recruitment strategy is commendably comprehensive; however, operational definitions of "under-represented groups" could be more explicit.*

**Response: **We have added particular demographics being monitored to support recruitment to a representative sample and identification in real-time of under-represented groups to operationally support recruitment strategy. The following has been added to the Participant Recruitment section to clarify this:

 ‘Demographics of participants recruited will be closely monitored across age, sex, disability, ethnicity and socioeconomic status to identify any under-represented groups against an expected representative sample calculated from UK hypertension prevalence data and UK population data. This will help guide targeting of under-represented groups and achieving a representative sample as discussed below.’

1. *Safety Considerations. The protocol rightly excludes individuals with contraindications. Including a brief summary of potential wall-squat-related risks (e.g., muscle strain, dizziness) would strengthen the safety section.*

**Response: **Wall squat related risks would not indicate a reason for exclusion from the study. This is because isometric exercise wall squats do not pose any risk to individuals over and above all forms of exercise recommended to maintain a healthy lifestyle (e.g. muscle strain, dizziness etc). Where there are risks to certain individuals with contraindications, for example those with pre-existing cardiovascular disease, these participants would be excluded under the current criteria. As per reviewer 1, we have given further detail about the exclusion criteria for any medical condition that may contraindicate participant inclusion but this does not include expected wall-squat risks – see response 1 to reviewer above.

 To detail expected wall squat related risks, we have added the following to the Adverse event monitoring section of the manuscript:

 ‘Common wall squat related adverse events would be similar in nature to other forms of recommended exercise (e.g. aerobic exercise or strength training) and may include muscle soreness after exercise, stress on joints (particularly the knees and ankles), dizziness during or after exercise, blood pressure spikes if holding of breath occurs during exercise (instructions to breath normally is including in the instructions to participants) and potential post-exercise hypotension. These will be recorded throughout the study.’

1. *Data Sharing Section: It may be helpful to specify whether anonymised qualitative data will also be available upon request.*

**Response: **This has been clarified in the data availability statement. Data that will be made available on request after the study includes quantitative study data and qualitative study data where the participants have opted in to the use of their anonymised data for future research during the consent process.

# Reviewer report: Reviewer response for version 1

**DOI:** 10.3310/nihropenres.15289.r37168

**Published:** 2025-10-07

**Authors:** Giuseppe Caminiti

**Affiliations:** 1 San Raffaele Open University, Rome; Cardiology Rehabilitation Unit, IRCCS San Raffaele, Rome, Italy

ISOFITTER study is a multi-centre randomized controlled trial designed to evaluate the effectiveness and real-world implementation of a simple isometric wall squat program for adults with stage 1–2 hypertension

 This is a well thought out study with potential iimplication in the daily management of blood pressure

 I only have few observations

 Methods

 Inclusion criteria: The sentence “…have no significant medical condition that would contraindicate their participation” is not clear enough and needs further explanations

 It is not clear to me why authors will exclude patients taking betablockers; this appears to be a major limitation: in fact the exclusion of patients on β-blockers will limit applicability of the study results to a substantial hypertensive subgroup.

 Authors should explain why the 12-months follow up will be restricted to only 50 subjects

Is the study design appropriate for the research question?

Yes

Is the rationale for, and objectives of, the study clearly described?

Yes

Are sufficient details of the methods provided to allow replication by others?

Yes

Are the datasets clearly presented in a useable and accessible format?

Yes

Reviewer Expertise:

Cardiac  rehabilitation

I confirm that I have read this submission and believe that I have an appropriate level of expertise to confirm that it is of an acceptable scientific standard.

# Response

**Published:** 2025-12-08

**Authors:** Melanie Rees-Roberts

**Affiliations:** CHSS, University of Kent, Canterbury, England, UK

Dear Reviewer,

 Many thanks for taking the time to read and comment on this article. the authors would like to thank you for your helpful and constructive comments in reviewing this protocol paper. The following changes have been made in response to your comments:

Reviewer 1 (7 Oct 2025)

  

1. *Methods: Inclusion criteria: The sentence “…have no significant medical condition that would contraindicate their participation” is not clear enough and needs further explanations*

**Response: **We have added the following to clarify a significant medical condition that would contraindicate participation:

 ‘A significant medical condition that would contraindicate participation outside of the exclusion criteria below is any medical condition declared by the participant or identified by the recommending physician which may affect a participant’s ability to undertake the intervention or their safety during the study (e.g. significant knee pain or weakness, epilepsy, narcolepsy, dementia etc).’

1. *It is not clear to me why authors will exclude patients taking betablockers; this appears to be a major limitation: in fact, the exclusion of patients on β-blockers will limit applicability of the study results to a substantial hypertensive subgroup.*

**Response: **We have added the following text into the Participant Eligibility Criteria section to clarify this exclusion choice:

 ‘We excluded potential participants who were taking beta adrenergic blocking agents (beta-blockers) because attenuated heart rate, blood pressure and haemodynamic responses would potentially undermine efficacy when prescribing this as well as other forms of exercise to lower resting blood pressure. Whilst this approach may exclude a proportion of people with treated hypertension, we think this would be relatively few. This is because our study only includes people on a single antihypertensive agent and beta-blockers are fourth line agents in the treatment of uncomplicated hypertension according to NICE guidelines (National Institute for Health and Care Excellence (2019) Hypertension in adults: diagnosis and management NG136. Available at: https://www.nice.org.uk/guidance/ng136).’

 Our approach is supported by a study by Jiao et al., demonstrating that the prescription of beta-blockers first-line for hypertension has fallen from 20.0% in 2001-2005 to 7.9% in the 2011+ era (Tianze Jiao, Robert W Platt, Antonios Douros, Kristian B Filion, Prescription Patterns for the Use of Antihypertensive Drugs for Primary Prevention Among Patients With Hypertension in the United Kingdom, American Journal of Hypertension, Volume 35, Issue 1, January 2022, Pages 42–53, https://doi.org/10.1093/ajh/hpab137). A further study by Johnson et al., published in 2025 showed that beta-blockers are prescribed as first line therapy in 1.6% of cases (Johnson K, Beradid S, Brophy JM, Platt RW, Renoux C. Trends in first-line monotherapy and combination therapy for hypertension in UK primary care. Br J Clin Pharmacol. 2025; 1-11. doi:10.1002/bcp.70271). Therefore, whilst selection criteria might exclude up to 20% of prevalent patients prescribed antihypertensives prior to 2005. We maintain that exclusion of people treated with beta-blockers because it has the potential to blunt the effect of isometric exercise, the prescription of beta-blockers as first (to third) line therapy for hypertension is not in line with NICE guidelines and in the recent era is very uncommonly used as a first line agent as a result.

1. *Authors should explain why the 12-months follow up will be restricted to only 50 subjects*

**Response: **The decision to restrict 12-month follow up to only 50 participants was purely pragmatic and would enable some exploration of longer term outcomes within the funding envelope and project timeline of 30 months whilst balancing overall value for money to the UK funder. We have added the following under the follow up assessments section to explain this:

 ‘This longer term, 12-month follow up is restricted to 50 participants due to the funding envelope and duration of 30 months which allows the research to explore longer term outcomes within limited time and funding whilst maintaining value for money.’
